# CD169^+^ macrophages regulate PD-L1 expression via type I interferon and thereby prevent severe immunopathology after LCMV infection

**DOI:** 10.1038/cddis.2016.350

**Published:** 2016-11-03

**Authors:** Namir Shaabani, Vikas Duhan, Vishal Khairnar, Asmae Gassa, Rita Ferrer-Tur, Dieter Häussinger, Mike Recher, Gennadiy Zelinskyy, Jia Liu, Ulf Dittmer, Mirko Trilling, Stefanie Scheu, Cornelia Hardt, Philipp A Lang, Nadine Honke, Karl S Lang

**Affiliations:** 1 Institute of Immunology, University Hospital Essen, University of Duisburg-Essen, Essen, Germany; 2 Department of Gastroenterology, Hepatology and Infectious Diseases, Heinrich-Heine-University Düsseldorf, Düsseldorf, Germany; 3 Department of Immunology and Microbial Science, The Scripps Research Institute, La Jolla, CA, USA; 4 Clinic for Primary Immunodeficiency, Medical Outpatient Unit and Immunodeficiency Laboratory, Department of Biomedicine, University Hospital, Basel, Switzerland; 5 Institute of Virology, University Hospital Essen, University of Duisburg-Essen, Essen, Germany; 6 Department of Infectious Disease, Union Hospital, Tongji Medical College, Huazhong University of Science and Technology, Wuhan, China; 7 Institute of Medical Microbiology and Hospital Hygiene, Heinrich-Heine-University Düsseldorf, Düsseldorf, Germany

## Abstract

Upon infection with persistence-prone virus, type I interferon (IFN-I) mediates antiviral activity and also upregulates the expression of programmed death ligand 1 (PD-L1), and this upregulation can lead to CD8^+^ T-cell exhaustion. How these very diverse functions are regulated remains unknown. This study, using the lymphocytic choriomeningitis virus, showed that a subset of CD169^+^ macrophages in murine spleen and lymph nodes produced high amounts of IFN-I upon infection. Absence of CD169^+^ macrophages led to insufficient production of IFN-I, lower antiviral activity and persistence of virus. Lack of CD169^+^ macrophages also limited the IFN-I-dependent expression of PD-L1. Enhanced viral replication in the absence of PD-L1 led to persistence of virus and prevented CD8^+^ T-cell exhaustion. As a consequence, mice exhibited severe immunopathology and died quickly after infection. Therefore, CD169^+^ macrophages are important contributors to the IFN-I response and thereby influence antiviral activity, CD8^+^ T-cell exhaustion and immunopathology.

Chronic viral infection is a serious health concern. Many viruses, such as human immunodeficiency virus (HIV), hepatitis B virus (HBV) and hepatitis C virus (HCV), lead to viral persistence and dysfunction of adaptive immunity.^1^ The persistence of HCV can lead to chronic liver inflammation, resulting in liver cirrhosis, liver steatosis, end-stage liver failure or hepatocellular carcinoma. Many of these clinical problems are related to the constant activity of cytotoxic CD8^+^ T cells. Therefore, exhaustion of CD8^+^ T cells may be essential for preventing severe immunopathology in chronic infections. Although mechanisms of exhaustion that involve inhibitory receptors have been thoroughly studied on the T-cell side, it has not yet been determined which cell types modulate the expression of the ligands for inhibitory receptors and thereby contribute to T-cell exhaustion during chronic viral infection. Identifying such mechanisms may help explain why some patients suffer from severe immunopathology during chronic infection, whereas others do not.

Type I interferon (IFN-I) plays a dual role during viral infection. On the one hand, it limits viral replication because it directly induces antiviral factors in the infected cell.^2, 3^ Consequently, the absence of the interferon-*α*/*β* receptor (IFNAR) promotes viral replication and can result in persistence of virus.^4, 5, 6^ On the other hand, sustained IFN-I signaling induces immunosuppressive mechanisms, including the production of interleukin-10 (IL-10) and the expression of programmed cell death ligand 1 (PD-L1).^7, 8, 9^ IL-10 and PD-L1 are important inhibitors of CD8^+^ T cells and thereby limit the function of virus-specific CD8^+^ T cells. Programmed cell death protein 1 (PD-1) is upregulated on all activated CD8^+^ T cells,^10^ a finding suggesting that the regulation of its ligand (PD-L1) determines the fate of virus-specific CD8^+^ T cells. Viral infection can upregulate PD-L1 expression by target cells, and this expression mediates the immune escape of these cells from killing by cytotoxic T lymphocytes (CTLs).^11^ How professional immune cells regulate PD-L1 expression during an ongoing infection is not well defined.

Here we report that during infection with lymphocytic choriomeningitis virus (LCMV), CD169^+^ macrophages prolong the IFN-I response that mediates antiviral activity. In addition, a prolonged IFN-I response induces PD-L1 expression in the liver. The absence of CD169^+^ macrophages reduces antiviral IFN-I activity and also limits PD-L1 expression. As a result, mice exhibit overwhelming viral replication in the absence of CD8^+^ T-cell exhaustion, and this replication results in severe immunopathology and death of mice.

## Results

### Depletion of CD169^+^ macrophages affects a subtype of F4/80^+^ macrophages in the liver and viral control

**Figure 1:**
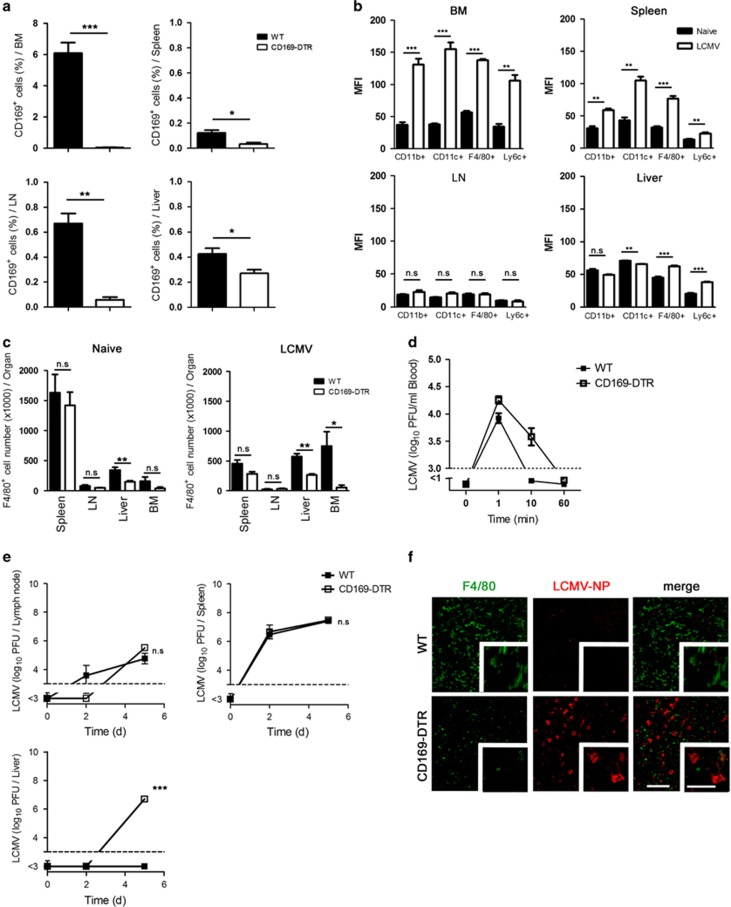
Depletion of CD169^+^ macrophages affects a subtype of F4/80^+^ macrophages in the liver and viral control. (**a**) Wild type (WT) and CD169 diphtheria toxin receptor (−DTR) mice were treated with diphtheria toxin (DT) day −3. On day 0, CD169^+^ cells were analyzed in the indicated organs (*n*=3). (**b**) Wild-type (WT) mice were infected with 30 plaque-forming units (PFU) of lymphocytic choriomeningitis virus strain WE (LCMV-WE) or left uninfected. On day 5, mean fluorescence intensity (MFI) of CD169 was measured in different organs (*n*=3). (**c**) Wild type (WT) and CD169 diphtheria toxin receptor (−DTR) mice were treated with diphtheria toxin (DT) day −3. On day 0 mice were infected with 30 PFU of LCMV-WE or left uninfected. On day 5, indicated organs were analyzed for F4/80^+^ cells (*n*=6 naive mice; *n*=3 LCMV-infected mice). (**d**) WT and CD169 diphtheria toxin receptor (−DTR) mice were treated with DT and infected intravenously with 2 × 10^6^ PFU LCMV-WE. Viral titers were measured in blood at indicated time points (*n*=6). (**e**) WT and CD169 diphtheria toxin receptor (−DTR) mice were treated with diphtheria toxin and infected intravenously with 30 PFU LCMV-WE. Viral titers were measured in various organs at indicated time points (*n*=3). (**f**) Wild type and CD169-DTR mice were treated with diphtheria toxin and infected intravenously with 30 PFU LCMV-WE. Liver sections collected 5 days after infection were stained for F4/80 (green) and LCMV nucleoprotein (−NP) (red) (*n*=3). Scale bars, 100 *μ*m (main images) or 50 *μ*m (insets). NS, not significant, *P<0.05, **P<0.01, ***P<0.001. Statistical significance was detected by Student's *t*-test (**a** and **c**) or analysis of variance (ANOVA) (**e**)

In order to study the role CD169^+^ cells, we used CD169 diphtheria toxin receptor (CD169-DTR) mice that express DTR under the CD169 promoter. Treating these mice with diphtheria toxin (DT) specifically depletes CD169^+^ cells. Interestingly, we found that after DT treatment, not only CD169^+^ cells in lymphoid organs were depleted but also the CD169^+^ cell number in the liver was reduced (Figure 1a and Supplementary Figure S1). Next we wondered on which cell type CD169 is expressed and whether its expression is upregulated during viral infection. To study this, we infected C57BL/6 wild type (WT) mice with LCMV strain WE and analyzed the expression of CD169 on different cell types in comparison with non-infected mice. We found that, without infection, CD169 is expressed on different cell types in the bone marrow and on F4/80^+^ cells in the liver and spleen (Supplementary Figure S2). After infection, CD169 was mostly upregulated in the bone marrow, on different cell populations in the spleen and on F4/80^+^ and Ly6C^+^ cells in the liver, whereas we did not detect an upregulation of CD169 in the lymph nodes (LNs) (Supplementary Figure S2 and Figure 1b). By analyzing the number of F4/80^+^ cells, we found a reduction in F4/80^+^ macrophages in the liver even in non-infected mice (Figure 1c). This means that a subtype of F4/80^+^ macrophages express CD169 in the liver under naive conditions. This raised the question whether depletion of CD169^+^ cells has an impact on phagocytic activity. We infected WT and CD169-DTR mice with LCMV-WE and measured viral titer in the blood. We detected more viruses in the blood of CD169-DTR mice after 10 min of infection in CD169-DTR mice (Figure 1d). However, virus was cleared from blood in both groups within 60 min post infection (Figure 1d). In a previous study, we showed that infecting WT mice with low dose of LCMV-WE (⩽200 PFU) leads to viral replication only in the spleen but not in the liver. The inhibition of viral replication in the liver was IFN-I-dependent because lack of interferon type I receptor led to high viral titer in the liver.^12^ Here we found that depletion of CD169^+^ macrophages in CD169-DTR mice did not affect viral replication in the spleen and LNs (Figure 1e). Also, after 5 days we could not detect enhanced viral replication (Figure 1e). However, CD169^+^ macrophages were essential for controlling LCMV replication in the liver 5 days after infection (Figure 1e). Histological staining showed that the presence of CD169^+^ macrophages in WT mice could prevent infection of hepatocytes, whereas mice without CD169^+^ macrophages exhibited virus-infected hepatocytes (Figure 1f). We conclude that depletion of CD169^+^ macrophages affects a subtype of F4/80^+^ macrophages in the liver which leads to enhanced viral replication.

### CD169^+^ macrophages in the spleen and LNs contribute to the production of IFN-I

**Figure 2:**
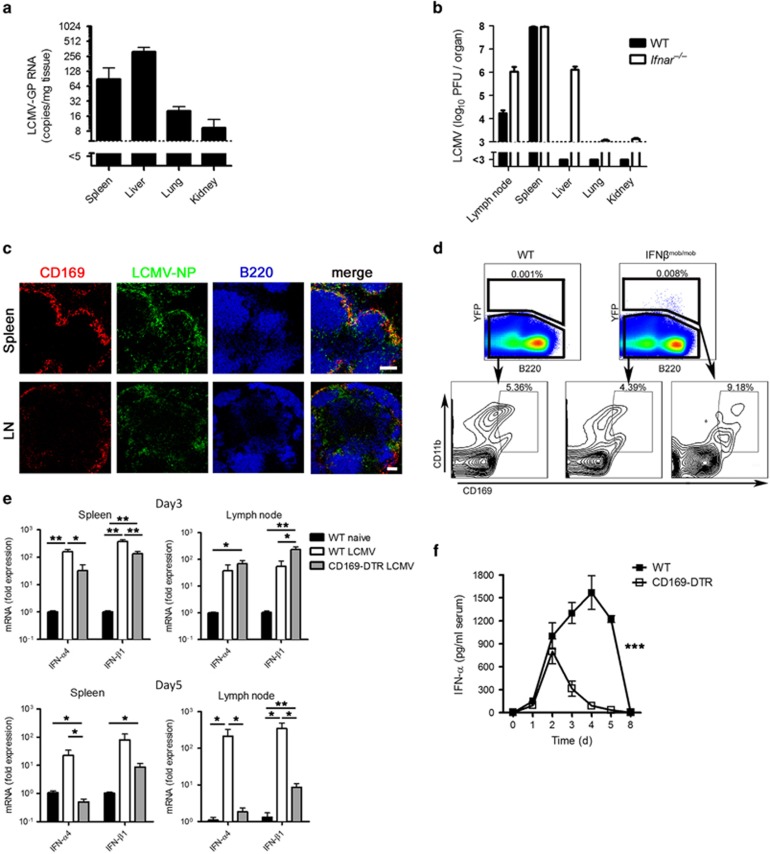
CD169^+^ macrophages in the spleen and lymph nodes contribute to the production of IFN-I. (**a**) Wild-type (WT) mice were infected intravenously with 2 × 10^6^ PFU (plaque-forming units) lymphocytic choriomeningitis virus strain WE (LCMV-WE). Viral RNA expression in different organs was analyzed after 60 min with quantitative real-time polymerase chain reaction (qRT-PCR) (*n*=6). (**b**) WT and interferon-*α*/*β* receptor–null (*Ifnar*^*–/–*^) mice were infected intravenously with 2 × 10^4^ PFU LCMV-WE. One day after infection, viral titers in various organs were measured (*n*=4). (**c**) WT mice were infected intravenously with 2 × 10^6^ PFU of LCMV-WE. Spleen sections collected 1 day after infection or lymph node (LN) sections collected 3 days after infection were stained for CD169 (red), LCMV nucleoprotein (NP) (green) and B220 (blue) (*n*=3). (**d**) WT and interferon-*β* (IFN*β*) reporter knock-in (IFN*β*^mob/mob^) mice were infected intravenously with LCMV-WE (30 PFU) for 48 h. IFN-*β* expression was analyzed by fluorescence-activated cell sorting (FACS) (*n*=3). (**e** and **f**) WT and CD169-DTR mice were treated with diphtheria toxin and infected intravenously with LCMV-WE (30 PFU). (**e**) IFN-*α*4 and IFN-*β*1 expression were measured with quantitative real-time polymerase chain reaction (qRT-PCR) at the indicated time points in spleen and LNs (*n*=3–4). (**f**) Levels of IFN-*α* in the serum were measured by enzyme-linked immunosorbent assay (ELISA) at the indicated time points (*n*=3–10). Scale bars, 100 *μ*m. *P<0.05, **P<0.01, ***P<0.001. Statistical significance was detected by Student's *t*-test (**e**) or analysis of variance (ANOVA) (**f**)

In a previous study we found that after viral infection with the cytopathic vesicular stomatitis virus (VSV), macrophages in liver are contributing mainly in taking up the virus. Even though viral replication is suppressed in the liver and allowed in CD169^+^ macrophages in the spleen and LNs in an IFN-I dependent manner.^13^ To define the role of CD169^+^ macrophages during infection with the noncytopathic LCMV, we first infected C57BL/6 wild-type (WT) mice with 2 × 10^6^ plaque-forming units (PFU) of LCMV strain WE and analyzed the viral uptake. After 1 h, viral RNA levels in various organs were analyzed. We found that the liver initially takes up most of the virus followed by the spleen (Figure 2a). However, one day after infection, viral replication was suppressed in the liver and allowed in the spleen and LNs (Figure 2b). This suppression was IFN-I dependent because infecting IFN-I receptor-deficient mice (*Ifnar*^*–/–*^) led to an increase in viral titer in the liver (Figure 2b). Histological staining of spleen and LNs showed that viral replication in spleen and LNs was mostly occurring in the CD169^+^ macrophages and that LCMV nucleoprotein (LCMV-NP) partially co-localized with CD169^+^ macrophages (Figure 2c). Because virus can induce IFN-I via activation of pattern recognition receptors,^14^ we hypothesized that replication of virus in CD169^+^ macrophages may lead to induction of IFN-I in these cells. To test this hypothesis, we infected IFN-*β* reporter knock-in mice (IFN*β*^mob/mob^ mice) with LCMV. In these mice the yellow fluorescent protein (YFP) is expressed after activation of the *ifnb* promoter. Additionally, we infected C57BL/6 mice as negative controls. Two days after LCMV infection, we analyzed the expression of YFP. Infection of IFN*β*^mob/mob^ mice led to the expression of YFP in 0.008% of all splenocytes (Figure 2d), a finding suggesting that these were the main IFN-I producers during LCMV infection.^15, 16^ WT mice did not exhibit YFP expression after LCMV infection (Figure 2d). Gating of IFN-I-producing cells showed that approximately 10% of IFN-I producers expressed CD169. Next we performed real-time polymerase chain reaction (RT-PCR) for the early IFN-I genes *Ifn-α4* and *Ifn-β1* in spleen and LNs to determine whether the depletion of CD169^+^ macrophages can influence IFN-I production. We found that LCMV infection induced strong expression of *Ifn-α4* and *Ifn-β1* mRNA in the spleen and LNs of WT mice (Figure 2e). DT-treated CD169-DTR mice exhibited significantly lower mRNA levels of *Ifn-α4* and *Ifn-β1* than WT mice, especially on day 5 after infection (Figure 2e). In line with these results, we found that systemic IFN-*α* levels were significantly lower in the serum of DT-treated CD169-depleted mice than in WT mice 2 days after infection (Figure 2f). In conclusion, we found that early replication of LCMV in CD169^+^ macrophages resulted in IFN-I production. Especially at later stages of infection, CD169^+^ macrophages were responsible for most of the systemic production of IFN-α.

### CD169^+^ macrophages have limited impact on CD8^+^ T-cell priming but are essential for controlling acute viral infection and prevention of immunopathology

**Figure 3:**
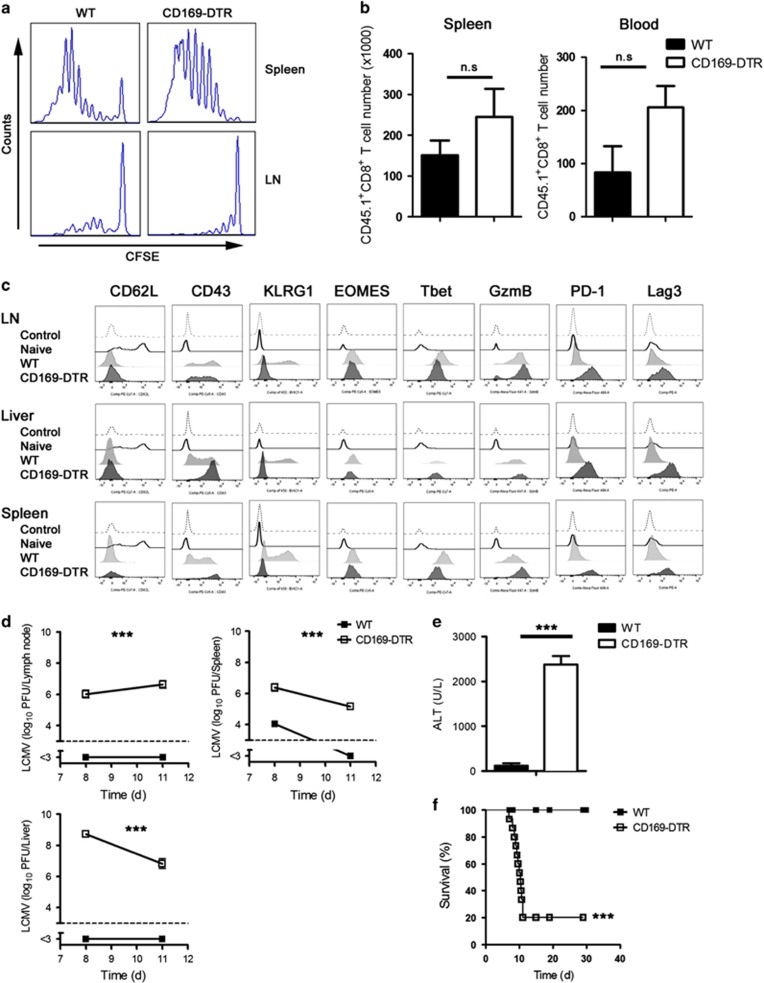
CD169^+^ macrophages have limited impact on CD8^+^ T-cell priming but are essential for controlling acute viral infection and prevention immunopathology. (**a** and **b**) Splenocytes (10^7^) from P14/CD45.1 mice were labeled with carboxyfluorescein succinimidyl ester (CFSE) and transferred to WT or CD169-DTR mice. On the next day, mice were infected with 30 PFU LCMV-WE. Four days after infection, (**a**) proliferation of CD45.1^+^CD8^+^ T cells was assessed by CFSE dilution in spleen and lymph nodes (LNs) (*n*=5). (**b**) Total number of CD45.1^+^CD8^+^ T cells in the spleen and blood 4 days after infection (*n*=5). (**c**) WT and CD169-DTR mice were treated with DT and infected intravenously with 30 PFU LCMV-WE. Indicated markers were measured in GP33-specific tetramer positive CD8^+^ T cells in LNs, liver and spleen 8 days after infection (*n*=3). (**d**–**f**) WT and CD169 diphtheria toxin receptor (−DTR) mice were infected intravenously with 30 PFU LCMV-WE. (**d**) Viral titers were measured in LNs, spleen and liver at indicated time points (*n*=3). (**e**) Alanine aminotransferase (ALT) activity in the serum was measured 8 days after infection (*n*=3). (**f**) Survival of WT and CD169-DTR mice was monitored (*n*=15). NS, not significant, ***P<0.001. Statistical significance was detected by Student's *t*-test (**b** and **e**), analysis of variance (ANOVA) (**d**) or log-rank (Mantel-Cox) test (**f**)

To determine whether early priming of CD8^+^ T cells is influenced by CD169^+^ macrophages, we transferred carboxyfluorescein succinimidyl ester (CFSE)-labeled splenocytes from naive CD45.1 × LCMV-P14 T-cell receptor transgenic mice (CD45.1 × P14 mice) into WT or CD169-DTR mice. P14 mice express a LCMV-GP33-41-specific TCR as a transgene.^17^ Infection with LCMV resulted in proliferation of virus-specific CD8^+^ T cells in the spleen and LNs in both WT and CD169-DTR mice (Figure 3a). The total number of virus-specific CD8^+^ T cells was slightly higher in the absence of CD169^+^ macrophages (Figure 3b). This finding suggests that CD169^+^ macrophages exert limited impact on CD8^+^ T-cell priming.

Next we investigated the activation markers of GP33-specific tetramer positive CD8^+^ T cells (Tet-GP33^+^). We treated WT and CD169-DTR mice with DT and infected them with LCMV-WE. We found, after 8 days of viral infection, that in CD169-DTR mice, Tet-GP33^+^ CD8^+^ T cells are more highly activated than in WT mice, as determined with granzyme B (GzmB), CD43, PD-1 and Lag3 (Figure 3c). Together, our findings suggest that the absence of CD169^+^ macrophages does not affect priming of virus-specific CD8^+^ T cells.

The absence of CD169^+^ macrophages was associated with a weak IFN-I response but normal CD8^+^ T-cell priming. We next investigated the impact of CD169^+^ macrophages on overall virus control and pathology after infection with an acute virus strain. We found that LCMV persists in LNs, spleen and liver of CD169-DTR mice but is controlled by WT mice (Figure 3d). This finding was in line with enhanced liver cell damage, because alanine aminotransferase (ALT) activity was dramatically increased in CD169-depleted mice (Figure 3e) and these mice became terminally ill after acute infection, whereas WT mice survived (Figure 3f). We conclude that absence of CD169^+^ cells has limited impact on T-cell priming but it leads to viral persistence and immunopathology.

### CD169^+^ macrophages induce PD-L1 expression which prevents immunopathology

**Figure 4:**
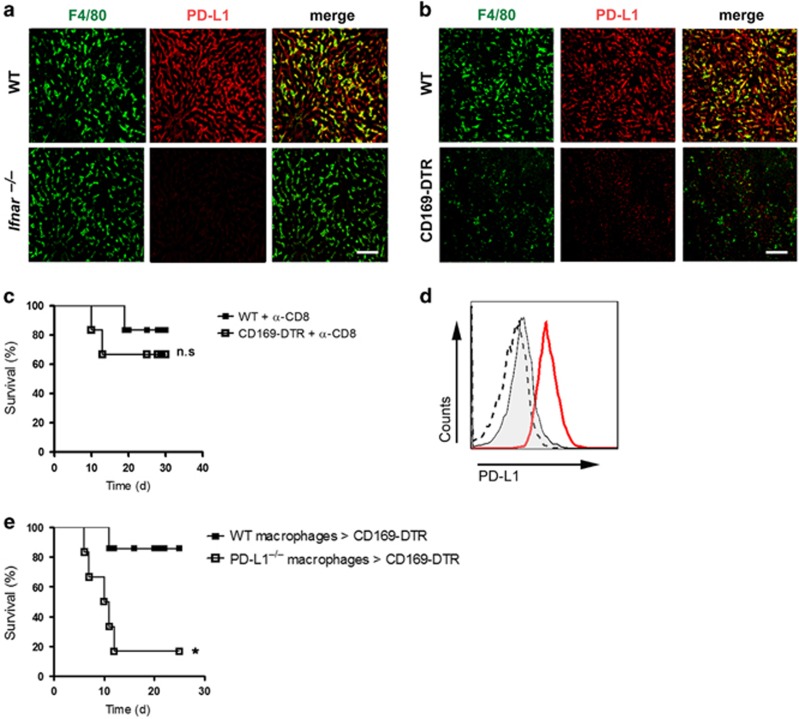
CD169^+^ macrophages induce PD-L1 expression which inhibits immunopathology. (**a**) Wild type (WT) and interferon-*α*/*β* receptor-null (*Ifnar*^*–/–*^) mice were infected intravenously with 30 PFU of lymphocytic choriomeningitis virus strain WE (LCMV-WE). Three days after infection, liver sections were stained for F4/80 (green) and PD-L1 (red) (*n*=3). Scale bar, 100 *μ*m. (**b**) WT and CD169 diphtheria toxin receptor (−DTR) mice were treated with diphtheria toxin and infected intravenously with 30 PFU LCMV-WE. Five days after infection, liver sections were stained for F4/80 (green) and PD-L1 (red) (*n*=3). Scale bar, 100 *μ*m. (**c**) Wild type (WT) and CD169 diphtheria toxin receptor (−DTR) mice were treated with DT and anti-CD8 antibody on days −1 and 3. Mice were infected intravenously with 30 PFU LCMV-WE and survival of WT and CD169-DTR mice was monitored (*n*=6). (**d**) Bone marrow-derived macrophages (BMDM) from wild type (WT; red line) or programmed cell death ligand 1-null mice (PD-L1^–/–^; dashed line) were treated overnight with 100 units of IFN-*α*4. Untreated WT macrophages were used as a control (filled line). Upregulation of PD-L1 was measured by fluorescence-activated cell sorting (FACS). (**e**) CD169-DTR mice were infected with 30 PFU LCMV-WE. 8 × 10^6^ BMDMs generated either from WT or from programmed cell death ligand 1-null mice (PD-L1^–/–^) were transferred into mice on days 5 and 7 post infection. Survival of mice was monitored (*n*=6). NS, not significant, *P<0.05. Statistical significance was detected by log-rank (Mantel-Cox) test (**c** and **e**).

IFN-I plays two main roles during LCMV infection. First, it directly inhibits viral replication by inducing antiviral enzymes. Second, it upregulates PD-L1 expression, and this upregulation can lead to exhaustion of CD8^+^ T cells. This finding suggested that the absence of CD169^+^ macrophages not only limits direct antiviral effects but may also influence CD8^+^ T-cell functions. The fact that the liver strongly responds to antiviral IFN-I, we investigated whether PD-L1 expression is regulated by IFN-I in the liver. We infected WT and *Ifnar*^*–/–*^ mice with LCMV and analyzed the expression of PD-L1 in the liver. We found that during LCMV infection, Kupffer cells express high levels of PD-L1 (Figure 4a). The absence of IFN-I signaling in *Ifnar*^*–/–*^ mice inhibits the upregulation of PD-L1 on Kupffer cells during acute infection (Figure 4a). Next we investigated whether CD169^+^ macrophages affect PD-L1 expression. To do so we stained the cells for PD-L1 in CD169-depleted mice after LCMV infection. PD-L1 expression was strongly reduced in the liver of DT-treated CD169-DTR mice (Figure 4b). This finding suggests that CD169^+^ macrophages-derived IFN-I is essential for PD-L1 upregulation in the liver.

We speculate that higher viral replication and a reduction of CD8^+^ T-cell exhaustion due to lack of PD-L1 expression in CD169-DTR mice are responsible for the death of the mice. Indeed depletion of CD8^+^ T cells prevented death in 70% of the CD169-DTR mice (Figure 4c). In order to compensate for PD-L1 expression in CD169-DTR mice, we generated bone marrow-derived macrophages (BMDMs) from WT and PD-L1-deficient mice and treated them with IFN-I in order to increase PD-L1 expression (Figure 4d). Afterwards, we transferred the BMDMs into two groups of LCMV-infected CD169-DTR mice. The group that received WT cells survived after infection with LCMV, whereas the group that received PD-L1–deficient cells died of infection (Figure 4e). In conclusion, the absence of CD169^+^ macrophages leads to a lethal immunopathology because of the limited expression of PD-L1.

### CD169^+^ macrophages prevent severe immunopathology during chronic viral infection

**Figure 5:**
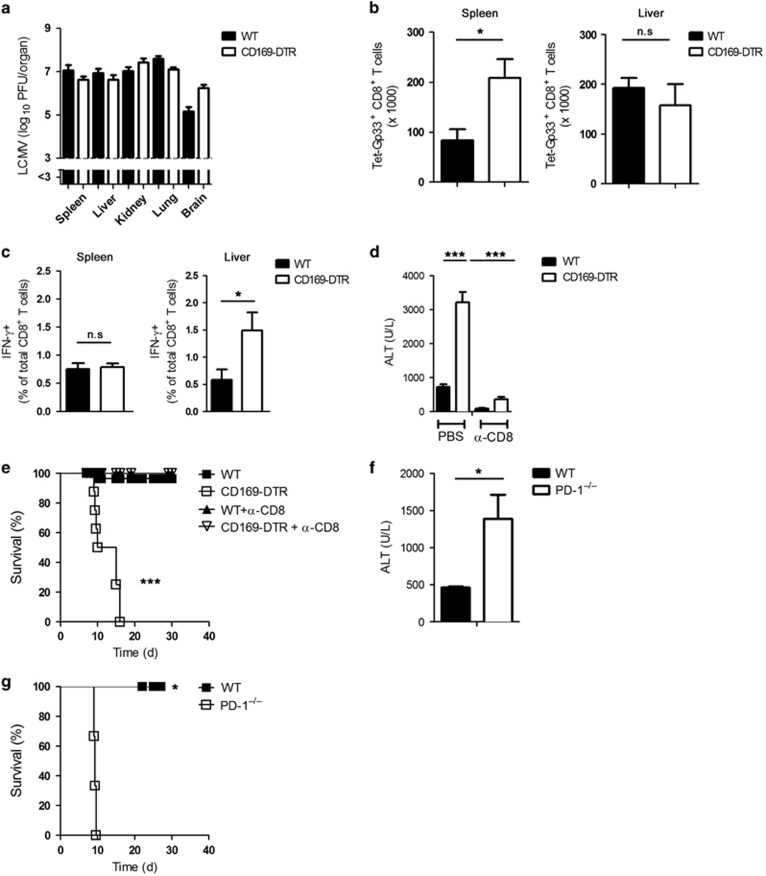
CD169^+^ macrophages prevent severe immunopathology during chronic viral infection. (**a**–**c**) Wild type (WT) and CD169 diphtheria toxin receptor (−DTR) mice were infected intravenously with 2 × 10^4^ plaque-forming units (PFU) of lymphocytic choriomeningitis virus strain Docile (LCMV-Docile) for 11 days. (**a**) Viral titers were measured in various organs (*n*=6). (**b**) Number of virus-specific Tet-GP33^+^ CD8^+^ T cells was determined in the spleen and liver (*n*=7–8). (**c**) IFN-*γ*^+^CD8^+^ T cells were counted in the spleen and liver (*n*=7–8). (**d**) WT and CD169-DTR mice were infected intravenously with 2 × 10^4^ PFU LCMV-Docile and treated with anti-CD8 depletion antibody or left untreated. Serum alanine aminotransferase (ALT) activity was measured after 13 days (*n*=3-4). (**e**) WT and CD169-DTR mice were infected intravenously with 2 × 10^4^ PFU LCMV-Docile and treated with anti-CD8 depletion antibody or left untreated. Survival of mice was monitored (*n*=8–33). (**f** and **g**) WT and programmed cell death protein 1-null (PD-1^–/–^) mice were infected intravenously with 2 × 10^4^ PFU LCMV-Docile. (**f**) Serum ALT activity was measured after 13 days (*n*=3). (**g**) Survival of mice was monitored (*n*=3). NS, not significant, *P<0.05, ***P<0.001. Statistical significance was detected by Student's *t*-test (**b**, **c**, **d** and **f**) or log-rank (Mantel-Cox) test (**e** and **g**)

We found that during acute LCMV infection, the absence of CD169^+^ macrophages results in insufficient control of virus and limited CD8^+^ T-cell exhaustion. Both effects result in severe immunopathology. Next we analyzed how CD8^+^ T-cell responses develop during overwhelming viral replication. To do so, we used the LCMV strain Docile, which is known to persist and is associated with severe CD8^+^ T-cell exhaustion in WT mice.^18^ In WT mice and CD169-DTR mice infected with LCMV-Docile, virus persisted after infection (Figure 5a). CD169-DTR mice generated more virus-specific CD8^+^ T cells in the spleen than did WT mice, whereas in both types of mice the numbers of these cells in the liver were comparable (Figure 5b). Moreover, CD8^+^ T cells in CD169-DTR mice exhibited higher frequencies of IFN-*γ* producing CD8^+^ T cells in the liver (Figure 5c). In the absence of CD169^+^ macrophages, liver cell damage was exacerbated (Figure 5d) and mice became terminally ill after infection (Figure 5e). To check whether this severe liver cell damage and clinical disease was dependent on CD8^+^ T-cell-mediated immunopathology, we depleted CD8^+^ T cells in both groups. We found that absence of CD8^+^ T cells in CD169-DTR mice prevented morbidity and mortality (Figures 5d and e). To determine whether the immunopathology in CD169-DTR mice was due to their inability to induce PD-L1 expression, we infected PD-1-deficient mice with LCMV-Docile. Indeed, the absence of PD-1 during chronic LCMV infection led to liver damage, as determined by serum ALT activity (Figure 5f); this phenotype resembled that of mice deficient in CD169^+^ cells (Figure 5d). The immunopathology also led to the death of LCMV-Docile-infected PD-1-deficient mice (Figure 5g). From these findings we concluded that CD169^+^ macrophages are essential for preventing severe immunopathology and death during chronic viral infection.

## Discussion

The results of this study show that CD169^+^ macrophages are important contributors to prolonged IFN-I production after LCMV infection. This prolonged production is associated with reduced viral replication in peripheral organs and with upregulation of PD-L1. Depletion of CD169^+^ macrophages results in enhanced viral propagation and prevents CD8^+^ T-cell exhaustion, both of which contribute to severe immunopathology.

The function of CD8^+^ T cells must be carefully regulated to ensure the elimination of virus without the development of severe immunopathology. IFN-I has been found to be a crucial innate cytokine that strongly regulates CD8^+^ T-cell function. First, it protects CD8^+^ T cells from cytotoxicity induced by natural killer cells.^19^ Second, IFN-I upregulates IL-10 and PD-L1, and this upregulation contributes to the exhaustion of CD8^+^ T cells.^8, 9^ Genetic deletion of IL-10 or partial blockade of PD-1 enhances the control of virus during chronic infection.^7, 20^ Earlier studies showed that PD-1^high^ CTLs are effective and important in reducing viral titers during acute infection.^10^ The factor that determines whether PD-1 expression can contribute to CD8^+^ T-cell exhaustion is the upregulation of PD-L1 on cells that contact CD8^+^ T cells.^11^ In addition to PD-1, 13 other inhibitory cell surface pathways have been shown to be overexpressed in exhausted CD8^+^ T cells.^21^ In addition to passive defects in metabolism, active suppression is needed for functional exhaustion of CD8^+^ T cells.^21^ The results of the present study show that mice lacking CD169^+^ macrophages cannot control LCMV because of the absence of prolonged production of IFN-I. Therefore, CD169^+^ macrophages may be the crucial cell type that balances viral suppression and CD8^+^ T-cell exhaustion.

In earlier studies using VSV, we found that CD169^+^ macrophages are essential for enforcing viral replication in the spleen and thereby play an important part in immune activation.^12, 13, 22^ In this study using LCMV, we did not find reduced priming of adaptive immune cells; however, we did find reduced induction of IFN-I. There may be two reasons for these diverse functions of CD169^+^ macrophages in VSV infection and LCMV infection. First, LCMV can replicate not only in follicular dendritic cells but also in CD169^+^ macrophages and conventional dendritic cells.^12^ Therefore, enforced viral replication does not depend entirely on CD169^+^ macrophages that will lead to early CD8^+^ T-cell activation and IFN-I production. Second, CD169^+^ macrophages in the liver may also participate in antiviral effector functions. Therefore, the absence of CD169^+^ macrophages may also limit local IFN-I production in the liver.

It remains to be answered how these findings can be transferred to chronic viral diseases in humans. The role of various subtypes of Kupffer cells during chronic viral infection has not been well studied. Some human macrophages express CD169.^23^ Whether these macrophage populations similarly contribute to the prolonged induction of IFN-I during chronic viral infections in humans remains to be studied.

In conclusion, we found that CD169^+^ macrophages contribute to viral propagation and CD8^+^ T-cell exhaustion during viral infection. CD169^+^ macrophage-derived IFN-I is essential for preventing viral replication in peripheral organs and for inducing PD-L1 expression so that severe immunopathology can be prevented.

## Materials and Methods

### Mice

All mice were sex, age and weight matched to their controls. CD169-DTR mice were generated in the Tanaka lab,^24^ and IFN*β*^mob/mob^ mice were generated in the Scheu lab.^16^ P14 mice expressing a LCMV-Gp33-41-specific TCR as a transgene were used for adoptive transfer experiments.^17^ All of these mice were maintained on a C57BL/6 background, as were PD-1^–/–^, PD-L1^–/–^ and *Ifnar*^*–/–*^ mice. All experiments were performed with the animals housed in single ventilated cages and with the authorization of the Veterinäramt Nordrhein Westfalen (Düsseldorf, Germany) in accordance with the German law for animal protection or the institutional guidelines of the Ontario Cancer Institute.

### Depletion of the cells

For CD8^+^ T-cell depletion, 500 *μ*g of anti-CD8 antibody clone YTS 169.4 (Bioxcell, West Lebanon, NH, USA) was injected intraperitoneally on days 1, 3 and 5. For the depletion of CD169^+^ macrophages, 30 *μ*g/kg body weight DT (Sigma Aldrich, St. Louis, MO, USA) was injected intraperitoneally on days −3, 2 and 5.

### Generation of bone marrow-derived macrophages and transfer experiment

Primary macrophages were generated by isolating bone marrow from femurs and tibias of mice and eliminating erythrocytes. Macrophages were generated by culturing bone marrow cells in very low endotoxin Dulbecco's modified Eagle's medium (VLE-DMEM) (Biochrom, Berlin, Germany) supplemented with 10% (v/v) fetal bovine serum (Biochrom), 0.1% (v/v) *β*-mercaptoethanol (*β*-ME) (Invitrogen, Carlsbad, CA, USA) and 10 ng/ml macrophage colony-stimulating factor (made in house). Cells were treated with 100 units of IFN-*α*4 (PBL Assay Science, Pistcataway, NJ, USA) on day 7 for 12 h. On day 8 of harvesting, cells were washed and 8 × 10^6^ cells were transferred into LCMV-infected mice. Cells transfer was performed twice on day 5 and day 7 of infection.

### Plaque assay

Virus titers were measured with a focus-forming assay as previously described.^25^

### Real-time polymerase chain reaction

Total RNA was extracted with Trizol (Life Technologies, Carlsbad, CA, USA). The RNA was reverse-transcribed into cDNA with the Quantitect Reverse Transcription Kit (Qiagen, Hilden, Germany). Gene expression analysis was performed with assays from Qiagen: glyceraldehyde 3-phosphate dehydrogenase (GAPDH; QT01658692), IFN-*α* (QT01774353) or IFN-*β* (QT00249662). Relative quantities (RQs) were determined with the equation RQ=2–ddCt.

### Lymphocyte transfer

Splenocytes from P14/CD45.1 mice were labeled with 1 *μ*M CFSE (Invitrogen) and injected intravenously into mice. One day later, mice were infected with LCMV-WE. The proliferation of P14 T cells was assessed in the spleen and LNs with CFSE dilution by flow cytometry.

### Flow cytometry

Tetramers were provided by the National Institutes of Health (NIH) Tetramer Facility (Emory University, Atlanta, GA, USA). Cells were stained with allophycocyanin (APC)-labeled GP-33 major histocompatibility complex class I tetramers (GP-33/H-2Db) for 15 min at 37 °C. After incubation, the samples were stained with anti-CD8 (BD Biosciences, San Diego, NJ, USA) for 30 min at 4 °C. Erythrocytes were then lysed with 1 ml BD lysing solution (BD Biosciences), washed once and analyzed by flow cytometry. Absolute numbers of GP-33-specific CD8^+^ T cells per microliter of blood were determined by fluorescence-activated cell sorting (FACS) analysis using fluorescent beads (BD Biosciences). IFN-*γ* was purchased from eBiosciences (San Diego, CA, USA).

### Enzyme-linked immunofluorescent assays

ELISAs for IFN-*α* were performed according to the manufacturer's protocol (PBL Assay Science).

### Histology

Histologic analyses used snap-frozen tissue. Sections were stained with anti-PD-L1 (eBioscience, CA, USA), anti-CD169 (AbD Serotec, Oxfordshire, UK), anti-CD45R (B220) (eBioscience, CA, USA), anti-F4/80 (eBioscience, CA, USA) or anti-LCMV-NP (made in-house).

### Alanine aminotransferase

Biochemical analyses were performed by the Central Laboratory, University hospital, Essen, Germany.

### Statistical analysis

Unless otherwise stated, data are expressed as mean±S.E.M. Student's *t*-test was used to detect statistically significant differences between groups. Significant differences between several groups were detected by two-way analysis of variance (ANOVA). The level of statistical significance was set at *P*<0.05, *P*<0.01 or *P*<0.001.

## Abbreviations

ALT: alanine aminotransferase
CFSE: carboxyfluorescein succinimidyl ester
CD169-DTR: CD169 diphtheria toxin receptor
CTL: cytotoxic T lymphocytes
GzmB: granzyme B
IL-10: interleukin-10
IFNAR: interferon-*α*/*β* receptor
LCMV: lymphocytic choriomeningitis virus
PFU: plaque-forming units
PD-L1: programmed death ligand 1
RT-PCR: real-time polymerase chain reaction
IFN-I: type I interferon
WT: wild type
VSV: vesicular stomatitis virus
