# Supplementary figures and images for: CD169+ macrophages regulate PD-L1 expression via type I interferon and thereby prevent severe immunopathology after LCMV infection
Source: Cell Death Dis. 2016 Nov 3;7(11):e2446–. doi: 10.1038/cddis.2016.350 (PMC5260878; doi:10.1038/cddis.2016.350)

# Supplementary Figure S1

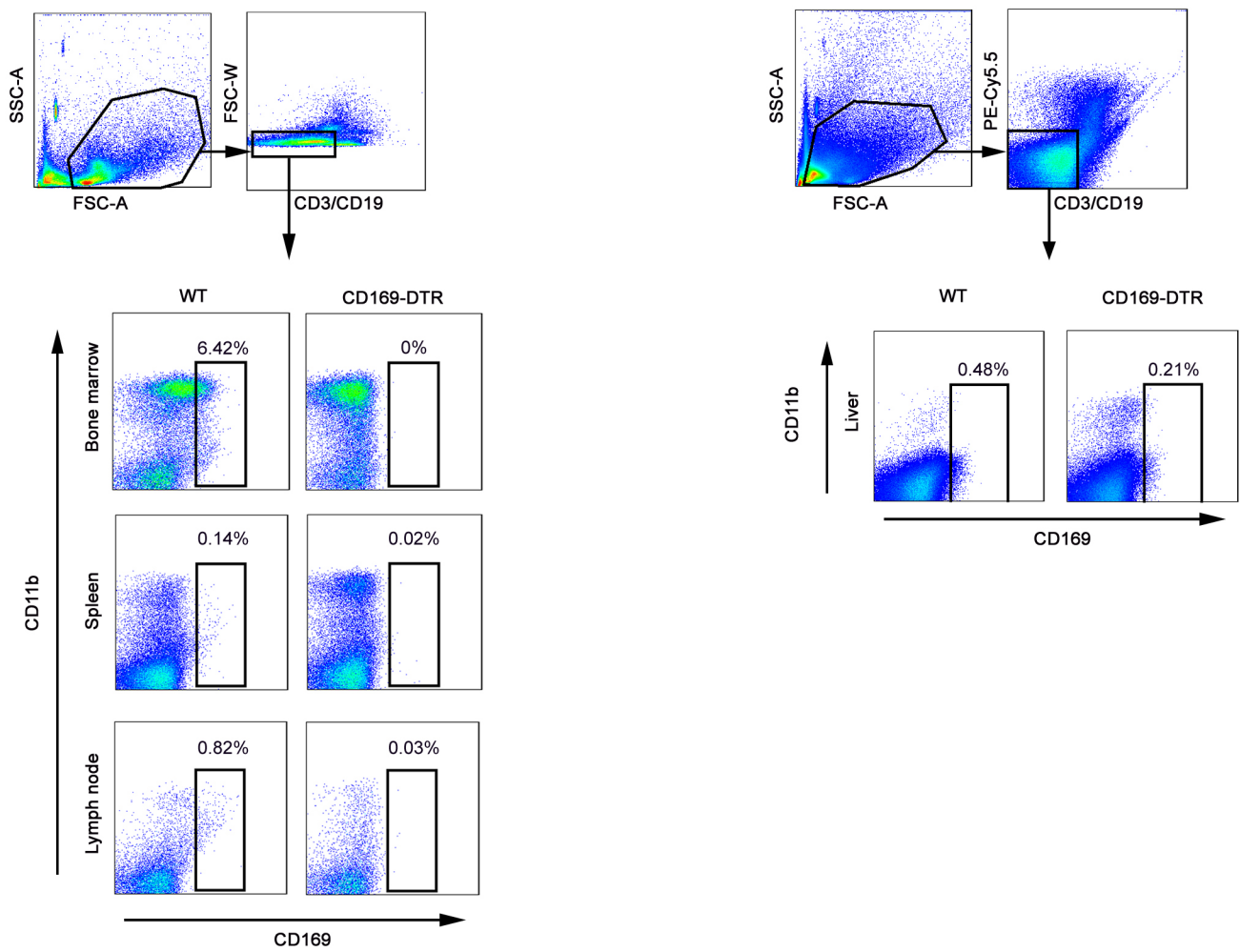

Supplement: Supplementary Figure 1 [file cddis2016350x1.pdf]

# Supplementary Figure S2

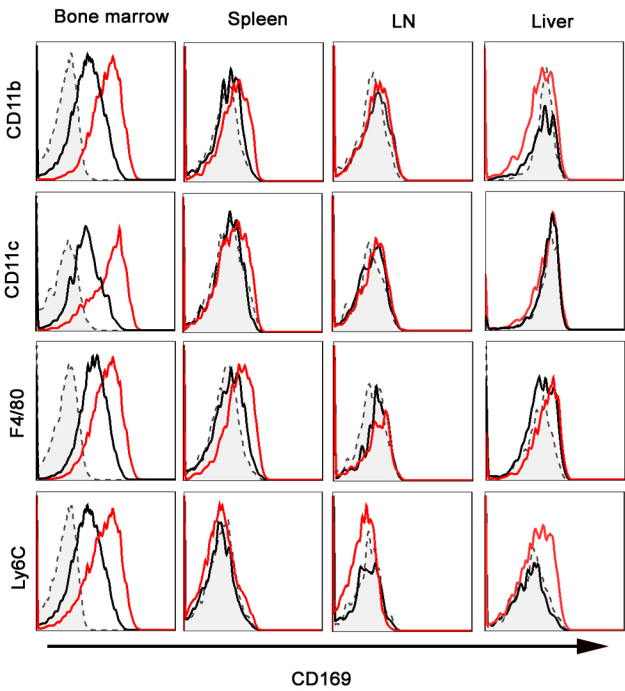

Supplement: Supplementary Figure 2 [file cddis2016350x2.pdf]
